# Time spent in moderate‑ to vigorous‑intensity physical activity is associated with lower limb muscle thickness in collegiate healthy young women without regular exercise habits

**DOI:** 10.3892/mi.2025.237

**Published:** 2025-04-30

**Authors:** Miho Anzai, Maya Hioki, Maria Sawa, Hitoshi Takahashi, Ryoma Michishita

**Affiliations:** 1 Graduate School of Sports and Health Science, Fukuoka University, Fukuoka 8140180, Japan; 2 Faculty of Health Care and Medical Sports, Department of Rehabilitation, Teikyo Heisei University, Chiba 2900170, Japan; 3 Shiodakinen Hospital, Chiba 2970203, Japan; 4 Faculty of Health Care and Medical Sports, Department of Medical Sports, Teikyo Heisei University, Chiba 2900170, Japan; 5 Faculty of Health and Sports Science, Fukuoka University, Fukuoka 8140180, Japan

**Keywords:** skeletal muscle, ultrasound tomography, the total energy expenditure, number of step counts, intramuscular adipose tissue

## Abstract

The present study aimed to investigate the association of physical activity and with or without regular exercise habits with lower limb muscle thickness, intramuscular adipose tissue (IntraMAT) and muscle strength in healthy young collegiate women, focusing on muscle thickness and IntraMAT using ultrasound tomography. The study participants included 20 healthy young collegiate women (age, 20.7±1.0 years). Physical activity was measured using a uniaxial accelerometer, and lower limb muscle thickness and IntraMAT were measured using ultrasound tomography. Muscle strength was measured as the maximum voluntary contraction force during isometric knee extension. The present study examined the association of physical activity with lower limb muscle thickness, IntraMAT and muscle strength, depending on whether the participants followed an exercise habit or not. Single correlation analysis revealed no significant correlation between the physical activity level and muscle thickness or muscle strength in all the participants. However, in the participants without regular exercise habits, the number of step counts and physical activity levels, particularly the time spent performing moderate- to vigorous-intensity activity, were positively associated with vastus lateralis muscle thickness (P=0.018 and P=0.048), and tended to have higher echo intensity of rectus femoris (P=0.056). These results suggest that a reduction in the time spent performing moderate- to vigorous-intensity physical activity may affect lower limb muscle thickness in healthy young collegiate women without regular exercise habits. In addition, the results suggest that a lack of exercise habits may influence the increase in IntraMAT in healthy young collegiate women.

## Introduction

Recently, several studies have reported a low physical activity among adult women worldwide (1-6). Lower levels of physical activity have developed into lifestyle-related diseases, such as obesity and type 2 diabetes. The reduction in physical activity has become that a social issue that needs to be solved in middle-aged, as well as older adults, but also in the younger generation. Some studies have reported that a loss of skeletal muscle mass and muscle strength was caused by long duration spent in inactivity, with more prominent effects observed in the lower extremities than in the upper extremities (7-9). Thus, long-term physical inactivity and lower levels of physical activity significantly affect the loss of skeletal muscle mass and muscle strength in the lower extremities.

Regular exercise habits and physical activity in young adults affect the age-related loss of skeletal muscle mass and muscle strength in middle-aged and older adults (10). Previous studies have suggested that peak values of skeletal muscle mass and muscle mass achieved in early adulthood may be an explanatory factor for the effects of age-related loss of skeletal muscle mass and strength (1). Several studies have shown that the regular exercise habits and physical activity levels in young adults determine whether an individual will be healthy during middle age and old age (10). Thus far, the rate of skeletal muscle mass loss with aging is greater in females than in males (1). In particular, it can be presumed that the formation of regular exercise habits in young adults to maintain and increase higher levels of physical activity is particularly critical for women to stay healthy during middle age and old age.

Ectopic adipose tissue, which accumulates within skeletal muscle [intramuscular adipose tissue (IntraMAT) accumulates due to aging (11-13). IntraMAT has also been implicated in physical impairments and the risk of falls (11). The factors affecting the IntraMAT content other than aging include blood properties, physical activity levels, physical performance and the dietary intake status (11). However, the association between these factors remains unclear. Among recent technological advancements, diagnostic imaging using ultrasound tomography has gained prominence, enabling the non-invasive and highly accurate evaluation of muscle mass, IntraMAT and subcutaneous fat thickness in addition to organs and blood vessels. It is widely used for various purposes from bedside use by trained clinicians to applications in sports fields (10).

However, to date, to the best of our knowledge, only a limited number of studies have evaluated muscle thickness and IntraMAT in young women using ultrasound tomography. The authors deemed that elucidating the association between physical activity levels and muscle thickness, evaluated using ultrasound tomography, may contribute to improving the health and increasing the physical activity of young women. Based on the hypothesis that physical activity levels in young women may be related to lower limb skeletal muscle thickness, IntraMAT and muscle strength, the present study aimed to investigate the association between physical activity levels and lower limb skeletal muscle thickness, IntraMAT and muscle strength in healthy young collegiate women.

## Subjects and methods

### Study participants

The present study included 20 healthy young collegiate women [mean age, 20.7±1.0 years; mean body mass index (BMI), 22.8±3.2 kg/m^2^; mean number of step counts, 7599.6±2338.9 counts]. Among the study participants, 12 participants did not have regular exercise habits and 8 participants had regular exercise habits, including 3 participants engaged in judo, 1 participant in soccer and 4 participants attending a sports course lecture. Patients with injuries to the lower extremities during the study period and those with a history of surgery, injury, or surgery during the past 6 months were excluded from the study. Prior to the study, the outline, purpose, risks, effectiveness associated with the study were explained to the participants, and written informed consent was obtained. The present study was approved by the Ethics Committee of Teikyo Heisei University (no. 2022-022-1) and was conducted in accordance with the guidelines of the Declaration of Helsinki.

### Anthropometry

The height, body weight, waist circumference and hip circumference of the participants were measured, and their BMI was calculated as the ratio of body weight (kg) to height squared (m^2^). The height and weight of the participants were measured to the nearest 0.1 cm or 0.1 kg while wearing light clothing. Height was measured using a metal stadiometer [Yoshida YS201-S(2M)]. Weight was measured using a scale (BS-302WT; Dretec). Waist and hip circumference were measured in the standing position using a tape measure. Waist circumference was measured at the level of the umbilicus and hip circumference was measured at the widest part of the hips.

### Assessment of physical activity levels

Physical activity levels were measured using a uniaxial accelerometer (Lifecorder EX; Suzuken Co. Ltd.). During the study, the participants were instructed to wear the accelerometer in a horizontal position for 16 consecutive days. Physical activity levels were measured for 14 days, excluding the first and last days. The measurements included the number of step counts, the total energy expenditure per day, energy expenditure during exercise and activity time categorized by intensity. Each participant wore an accelerometer on a belt at the waist level during the study period. Accelerometers were worn on the lower back from the time of waking up until bedtime, and removed during sports activities, bathing and swimming. Participants whose measurements were obtained for at least 10 out of the 14 days were included in the analysis, and the data were averaged for the 10-14-day period. The accelerometer used in the present study could determine the activity level from 1 to 9. Kumahara *et al* (14) reported a high correlation between energy expenditure calculated from this accelerometer and that measured using a metabolic chamber system, which is widely used in Japan. These physical activity levels were broadly classified as light [levels 1-3; ≥1.5 to <3 metabolic equivalents (MET)], moderate (levels 4-6; ≥3 to <6 MET) and vigorous intensity (levels 7-9; ≥6 MET), according to a previous study (14).

### Ultrasonic measurement of muscle thickness

Lower limb muscle thickness was measured using ultrasound tomography. Ultrasound tomography and data analysis were performed by a single investigator. All the participants refrained from participating in intense sports for 2 days prior to the ultrasound tomography. The system-setting parameters for ultrasound tomography (LOGIQ e V2; Cytiva) were as follows: B-mode; frequency, 8.0 MHz; gain, 80 dB; and depth, 7 cm. Imaging was performed with the participants placed in the prone position with the legs fully extended and relaxed. The ultrasound tomography positions were marked on the body surface in the dorsal recumbent and knee extension (0˚) positions using a glide meter (Martin Anthropometer; Takei Scientific Instruments) and measuring tape. The mid-thigh (midway between the greater trochanter and knee joint cleft) were measured and marked on the anterior and lateral sides of the mid-section. Measurements for the rectus femoris and vastus intermedius were obtained from the anterior part of the thigh and those for the vastus lateralis and vastus intermedius were obtained from the lateral part of the thigh. The rectus femoris and vastus lateralis were defined as the superficial and ventral fascia of each muscle, respectively, and the vastus intermedius was defined as the area between the fascia and femur. Subcutaneous tissue thickness was measured between the uppermost part of the skin and the superficial fascia of the muscle at the anterior and lateral sites. A total of five images were scanned for each section of the thigh and were averaged for future analysis. Ultrasound tomography and data analysis were performed as previously described (15). To minimize the difference in body size, muscle thicknesses of the rectus femoris and vastus intermedius were normalized by thigh length (muscle thickness per thigh length).

### Ultrasonic measurement of intramuscular fat measurement by echo intensity

IntraMAT was measured using ultrasound tomography. As the IntraMAT content estimated from echo intensity is consistent with magnetic resonance imaging and 1H-magnetic resonance spectroscopy, the intramuscular fat content was measured based on the ultrasound echo intensity. Echo intensity was analyzed using ImageJ software (version 1.44; National Institutes of Health). The region of interest (ROI) was selected to include muscle as much as possible with reference to a previous study (12), and bone and surrounding fascia were excluded (12). The mean echo intensity of the ROI was calculated (8-bit resolution, resulting in a value between 0 and 255; scale: black=0; white=255). All ultrasound tomography images of the rectus femoris, vastus lateralis and vastus intermedius muscles were analyzed using ImageJ software (version 1.53e; National Institutes Health), as previously described (12,13,16).

### Measurement of muscle strength

The maximal voluntary contraction (MVC) force during isometric knee extension was measured as the muscle strength of the right lower limb. Measurements were obtained with the knee joint flexed at 90˚ (full knee joint extension=0˚), using a measuring table for knee extension and flexion and a tension meter (T.K.K. 5715a, T.K.K. 5710e; Takei Scientific Instruments). Muscle contraction was sustained for 3 sec, with a 2-min rest period. The measurements were performed three times, and the average of the three measurements was used. Isometric knee extension force is expressed as an absolute value (Nm). To minimize the difference in body size, muscle strength was normalized by weight (muscle strength per weight). Muscle strength measurement and data analysis were performed according to previous studies (17).

### Statistical analysis

All data are expressed as the mean ± standard deviation. Comparisons of variables between the two groups were performed using the unpaired t-test, and Pearson's single correlation was used for associations between continuous variables. All statistical analyses were performed using SPSS software (version 23.0 J; SPSS IBM Corp.). Values of P<0.05 were considered to indicate statistically significant differences.

## Results

The physical characteristics of the participants are presented in Table I. The accelerometers were worn for an average of 13.3±0.7 days (10-14 days). The mean total energy expenditure, number of step counts, and time spent in inactivity, light-intensity, and moderate- to vigorous-intensity activity during the study period were 1791.4±152.7 kcal/day, 7599.6±23 counts, 1363.1±22.9 min/day, 50.3±17.2 min/day and 25.2±11.8 min/day, respectively. The mean muscle thickness of the vastus lateralis and rectus femoris was 2.11±0.30 and 1.72±0.32 cm, respectively.

**Figure 1:**
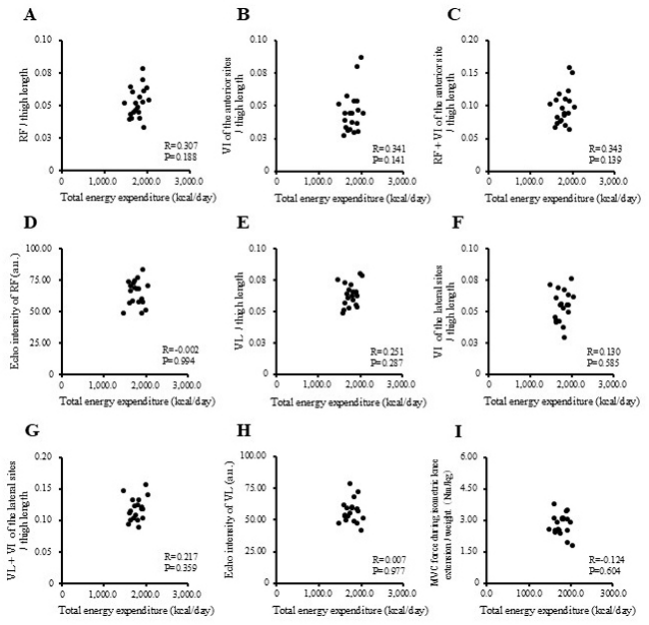
Correlation of total energy expenditure with muscle thickness normalized to thigh length, and muscle strength in all the participants. (A) RF per thigh length. (B) VI of the anterior sites per thigh length. (C) RF + VI of the anterior site per thigh length. (D) Echo intensity of RF (a.u.). (E) VL per thigh. (F) VI of the lateral sites per thigh length. (G) VL + VI of the lateral sites per thigh. (H) Echo intensity of VL (a.u.). (I) MVC force during isometric knee extension per weight (Nm/kg). RF, rectus femoris; VI, vastus intermedius; VL, vastus lateralis; MVPA, moderate-to-vigorous-intensity physical activity; MVC, maximal voluntary contraction.

**Figure 2:**
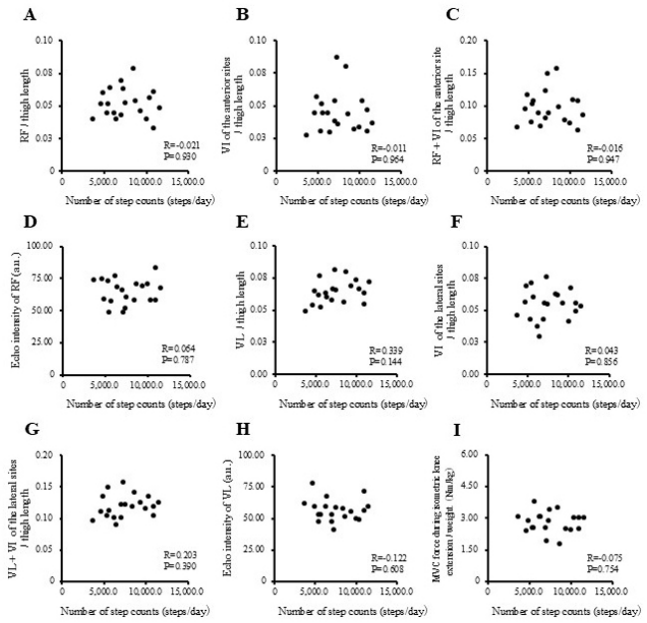
Correlation of number of step counts with muscle thickness normalized to thigh length, and muscle strength in all the participants. (A) RF per thigh length. (B) VI of the anterior sites per thigh length. (C) RF + VI of the anterior site per thigh length. (D) Echo intensity of RF (a.u.). (E) VL per thigh. (F) VI of the lateral sites per thigh length. (G) VL + VI of the lateral sites per thigh. (H) Echo intensity of VL (a.u.). (I) MVC force during isometric knee extension per weight (Nm/kg). RF, rectus femoris; VI, vastus intermedius; VL, vastus lateralis; MVPA, moderate-to-vigorous-intensity physical activity; MVC, Maximal voluntary contraction.

**Figure 3:**
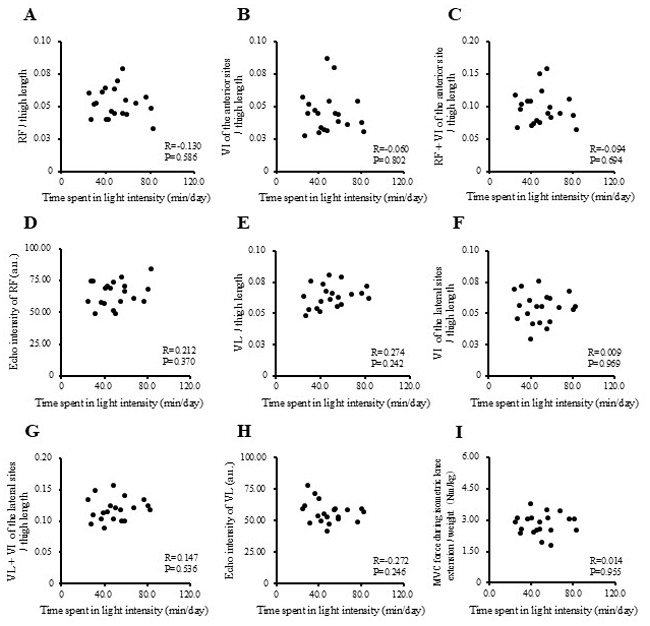
Correlation of time spent in light intensity physical activity with muscle thickness normalized to thigh length, and muscle strength in all the participants. (A) RF per thigh length. (B) VI of the anterior sites per thigh length. (C) RF + VI of the anterior site per thigh length. (D) Echo intensity of RF (a.u.). (E) VL per thigh. (F) VI of the lateral sites per thigh length. (G) VL + VI of the lateral sites per thigh. (H) Echo intensity of VL (a.u.). (I) MVC force during isometric knee extension per weight (Nm/kg). RF, rectus femoris; VI, vastus intermedius; VL, vastus lateralis; MVPA, moderate-to-vigorous-intensity physical activity; MVC, maximal voluntary contraction.

**Figure 4:**
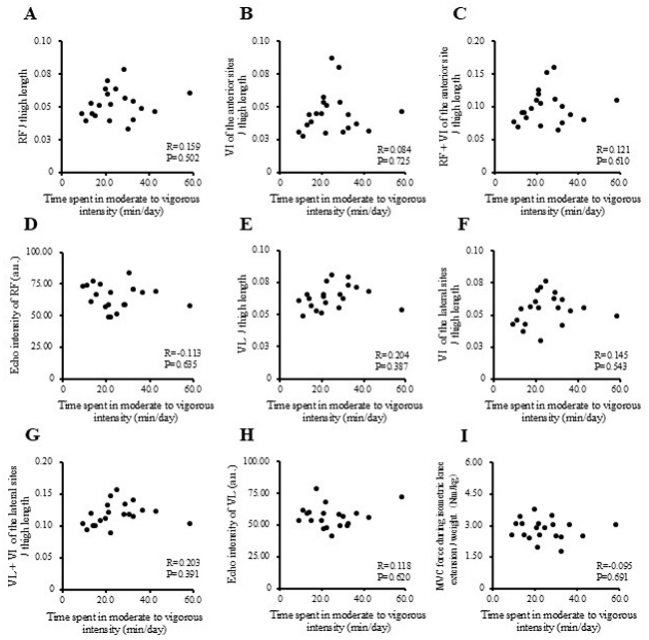
Correlation of time spent in moderate-to-vigorous-intensity physical activity with muscle thickness normalized to thigh length, and muscle strength in all the participants. (A) RF per thigh length. (B) VI of the anterior sites per thigh length. (C) RF + VI of the anterior site per thigh length. (D) Echo intensity of RF (a.u.). (E) VL per thigh. (F) VI of the lateral sites per thigh length. (G) VL + VI of the lateral sites per thigh. (H) Echo intensity of VL (a.u.). (I) MVC force during isometric knee extension per weight (Nm/kg). RF, rectus femoris; VI, vastus intermedius; VL, vastus lateralis; MVPA, moderate-to-vigorous-intensity physical activity; MVC, maximal voluntary contraction.

The correlations between physical activity levels with muscle thickness and muscle strength in the study participants are presented in Fig. 1, Fig. 2, Fig. 3 and Fig. 4 and Table II. Single correlation analysis did not reveal any significant correlation between the physical activity level and muscle thickness or muscle strength (Fig. 1, Fig. 2, Fig. 3 and Fig. 4 and Table II).

The differences in the physical characteristics of participants with or without regular exercise habits are presented in Table III. The present study aimed to clarify whether physical activity levels, muscle thickness and muscle strength were influenced by regular exercise habits. The group without regular exercise habits had significantly lower values of rectus femoris muscle thickness per thigh length (P=0.009), rectus femoris and vastus intermedius muscle thickness at the anterior site per thigh length (P=0.011) and muscle strength per weight (P<0.001). This group also trended to have a higher echo intensity of the rectus femoris (a.u.) (P=0.056) compared with the group with regular exercise habits (Table III).

**Figure 5:**
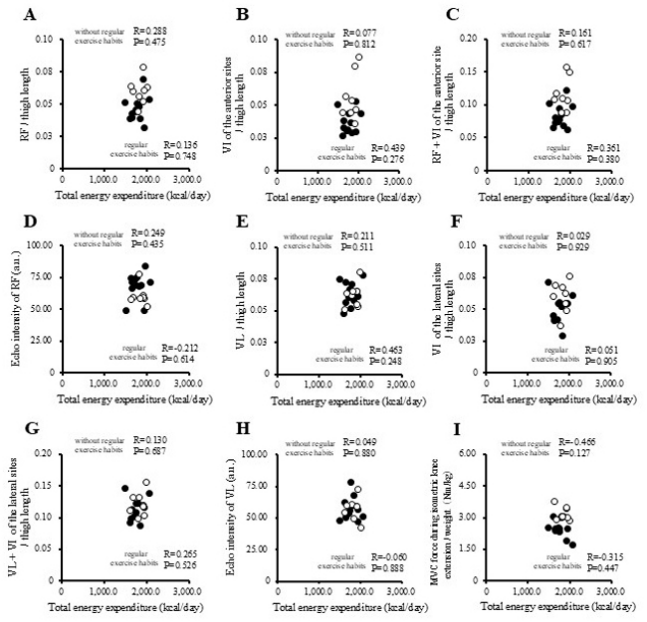
Correlation of total energy expenditure with muscle thickness normalized to thigh length, and muscle strength in with and without regular exercise habits healthy young collegiate women. White circles represent those with regular exercise habits and black circles represent those without regular exercise habits. (A) RF per thigh length. (B) VI of the anterior sites per thigh length. (C) RF + VI of the anterior site per thigh length. (D) Echo intensity of RF (a.u.). (E) VL per thigh. (F) VI of the lateral sites per thigh length. (G) VL + VI of the lateral sites per thigh. (H) Echo intensity of VL (a.u.). (I) MVC force during isometric knee extension per weight (Nm/kg). RF, rectus femoris; VI, vastus intermedius; VL, vastus lateralis; MVPA, moderate-to-vigorous-intensity physical activity; MVC, maximal voluntary contraction.

**Figure 6:**
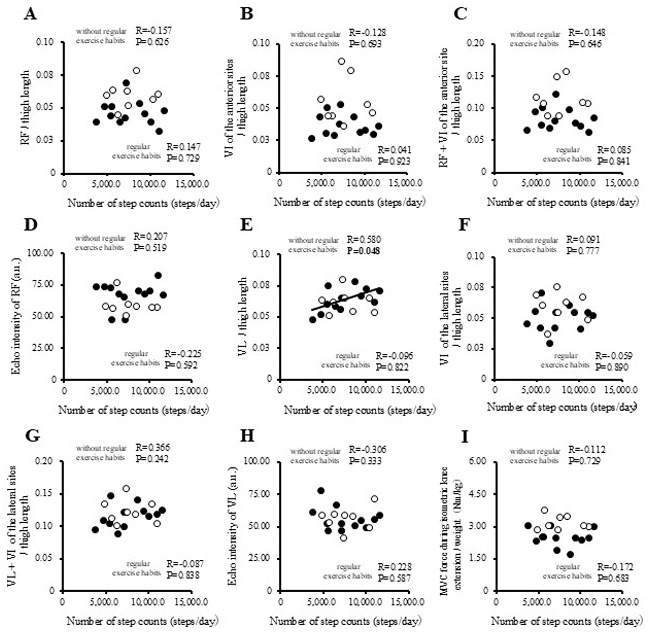
Correlation of number of step counts with muscle thickness normalized to thigh length, and muscle strength in with and without regular exercise habits healthy young collegiate women. White circles represent those with regular exercise habits and black circles represent those without regular exercise habits. (A) RF per thigh length. (B) VI of the anterior sites per thigh length. (C) RF + VI of the anterior site per thigh length. (D) Echo intensity of RF (a.u.). (E) VL per thigh. (F) VI of the lateral sites per thigh length. (G) VL + VI of the lateral sites per thigh. (H) Echo intensity of VL (a.u.). (I) MVC force during isometric knee extension per weight (Nm/kg). RF, rectus femoris; VI, vastus intermedius; VL, vastus lateralis; MVPA, moderate-to-vigorous-intensity physical activity; MVC, maximal voluntary contraction.

**Figure 7:**
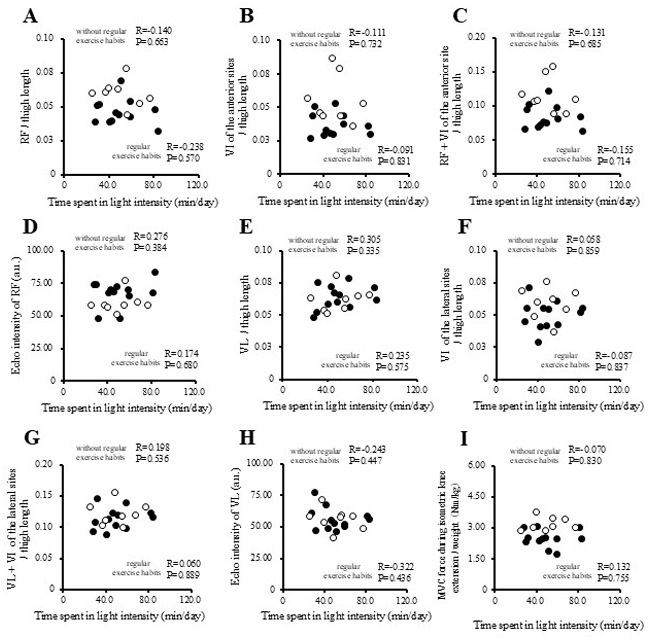
Correlation of time spent in light intensity physical activity with muscle thickness normalized to thigh length, and muscle strength in with and without regular exercise habits healthy young collegiate women. White circles represent those with regular exercise habits and black circles represent those without regular exercise habits. (A) RF per thigh length. (B) VI of the anterior sites per thigh length. (C) RF + VI of the anterior site per thigh length. (D) Echo intensity of RF (a.u.). (E) VL per thigh. (F) VI of the lateral sites per thigh length. (G) VL + VI of the lateral sites per thigh. (H) Echo intensity of VL (a.u.). (I) MVC force during isometric knee extension per weight (Nm/kg). RF, rectus femoris; VI, vastus intermedius; VL, vastus lateralis; MVPA, moderate-to-vigorous-intensity physical activity; MVC, maximal voluntary contraction.

**Figure 8:**
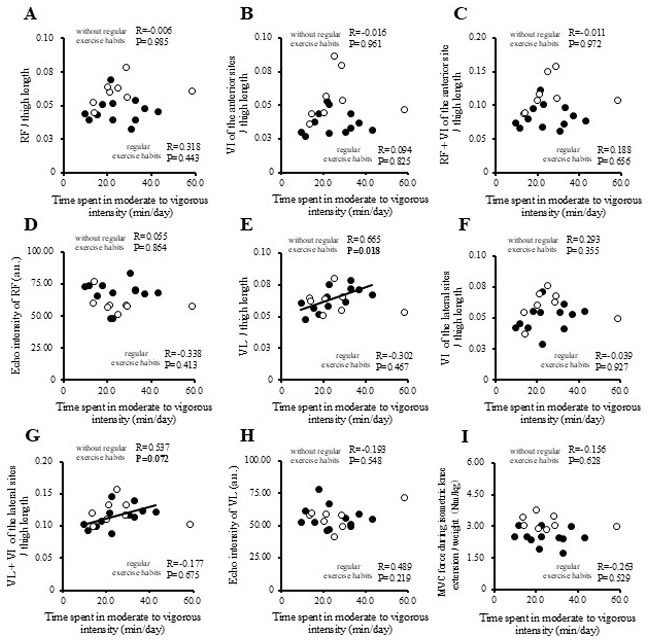
Correlation of time spent in moderate-to-vigorous-intensity physical activity with muscle thickness normalized to thigh length, and muscle strength in with and without regular exercise habits healthy young collegiate women. White circles represent those with regular exercise habits and black circles represent those without regular exercise habits. (A) RF per thigh length. (B) VI of the anterior sites per thigh length. (C) RF + VI of the anterior site per thigh length. (D) Echo intensity of RF (a.u.). (E) VL per thigh. (F) VI of the lateral sites per thigh length. (G) VL + VI of the lateral sites per thigh. (H) Echo intensity of VL (a.u.). (I) MVC force during isometric knee extension per weight (Nm/kg). RF, rectus femoris; VI, vastus intermedius; VL, vastus lateralis; MVPA, moderate-to-vigorous-intensity physical activity; MVC, maximal voluntary contraction.

The correlations between physical activity levels and muscle thickness and muscle strength in the groups with and without regular exercise habits are illustrated in Fig. 5, Fig. 6, Fig. 7 and Fig. 8 and Table IV. Of note, white circles represent the group with regular exercise habits, and black circles represent the group without regular exercise habits. In Fig. 5, Fig. 6, Fig. 7 and Fig. 8, some similarities may appear between the locations of the dots with those shown in Fig. 1, Fig. 2, Fig. 3 and Fig. 4. Fig. 1, Fig. 2, Fig. 3 and Fig. 4 include all the study participants, and Fig. 5, Fig. 6, Fig. 7 and Fig. 8 include all the study participants divided into two groups. In the group with regular exercise habits, no significant correlation was observed between the physical activity level and muscle thickness or muscle strength (Fig. 5, Fig. 6, Fig. 7 and Fig. 8). In the group without regular exercise habits, physical activity levels, particularly the time spent in moderate- to vigorous-intensity activity, positively correlated with vastus lateralis muscle thickness per thigh length (R=0.665, P=0.018; Fig. 8E and Table IV); a positive correlation was also found between the time spent in moderate to vigorous intensity and vastus lateralis and vastus intermedius muscle thickness at the lateral site per thigh length (R=0.537, P=0.072; Fig. 8G and Table IV), as well as between the number of step counts and the vastus lateralis muscle thickness at the lateral site per thigh length muscle thickness (R=0.580, P=0.048; Fig. 6E and Table IV). However, ImtraMAT was not significantly correlated with any of the physical activity levels and muscle thickness or muscle strength in the study participants.

## Discussion

The major findings of the present study were that the number of step counts and time spent performing moderate- to vigorous-intensity activity were positively associated with vastus lateralis muscle thickness at the lateral site per thigh length in the group without regular exercise habits. These results support the hypothesis that physical activity levels in young women may be related to lower limb muscle thickness and strength. It has been found that compared to those with moderate-to-vigorous-intensity physical activity (MVPA), individuals with lower physical activity levels exhibited significantly lower skeletal muscle mass index, and this association was observed in older adults, as well as in younger women (1,18-20). Oshita and Myotsuzono (1) reported that current physical activity among female students particularly affects the lower limb muscle mass. They demonstrated that physical inactivity or low physical activity may cause difficulty in independent mobility in the younger generation, even among those with regular exercise habits in the past (1). Previous studies have reported that 30-50% of healthy young Japanese women already fall under the cut-off value for skeletal muscle mass, a precursor to sarcopenia (1,21,22). Therefore, to maintain skeletal muscle mass, time spent in MVPA needs to be continued by the younger generation. Another study reported that the cross-sectional area (CSA) of the vastus lateralis of the quadriceps muscles decreased by ~40% in individuals aged between 20 and 80 years of age (23). The loss in the size of the quadriceps with age may contribute to low physical activity in habitual sports and physical activity, which require higher exercise intensities than most daily activities (24-26). Thus, based on the results of the present study and previous studies (1,21,22), maintaining lower limb muscle thickness and continuous engagement in MVPA are required to maintain and improve future health, even in healthy young women.

The results of the present study did not reveal any significant association between the physical activity level and muscle thickness or strength in the group with regular exercise habits. However, in the participants without regular exercise habits, physical activity levels, particularly the time spent in moderate- to vigorous intensity activity, were positively associated with vastus lateralis muscle thickness at the lateral site per thigh length. In previous research, the relative weight percentage of lower limb muscle thickness indicated that the thigh muscle group accounts for approximately half (46.9%) of the total skeletal muscle mass, with the quadriceps muscle accounting for the highest percentage (27). Furthermore, the CSA of the quadriceps muscle is composed of four muscles, of which the vastus lateralis occupies the largest physiological area, at ~30% (26,27). Jacob *et al* (28) also reported an association between the functional capability of lower limb muscle thickness in healthy adults aged 18-70 years during the course of daily life and the morphology of vastus lateralis muscle thickness. These findings indicate that vastus lateralis muscle thickness is negatively associated with age, and this association may be caused by the intensity and frequency of physical activity (28). Young adults engage in more vigorous-intensity weight shifts compared to older adults, while older adults engage in more light-intensity weight shifts compared to younger individuals. Physical activity of light- and vigorous-intensity is inferred from the types of mobilized muscles, and the difference in energy expenditure. The results of the present study did not reveal any significant association between the physical activity level and muscle thickness or strength in the group with regular exercise habits. The group with regular exercise habits practiced vigorous-intensity competitive sports in daily life and maintained vastus lateralis muscle thickness. Therefore, physical activities other than competitive sports may have been unaffected.

The results of the present study also revealed no significant association between the physical activity level and echo intensity in the study participants. However, in the participants without regular exercise habits, a higher echo intensity of the rectus femoris (a.u.) was found compared to the participants with regular exercise habits. Therefore, it was suggested that the continuation of exercise affects IntraMAT. Previous research has suggested that skeletal muscle size is a critical factor for the regulation of IntraMAT content and that increases in metabolic capacity of already existing muscles contribute to decreases in the IntraMAT content (29). Even among young women, continuous exercise is associated with an decreased IntraMAT, regardless of the amount of physical activity. These findings suggest that it may be crucial to establish regular exercise habits to prevent an increase in IntraMAT in young women.

In a previous study, moderate-intensity exercise in older adults in the exercise group was associated with significantly higher vastus lateralis CSA and intracellular resistance index scores on segmental bioelectrical impedance spectroscopy exercise than those of the older adults without regular exercise habits (30). However, in the present study, physical activity levels, particularly the time spent performing moderate- to vigorous-intensity activity by the participants without regular exercise habits were significantly and positively associated with vastus lateralis muscle thickness at the lateral site per thigh length. The group without regular exercise habits may have exhibited a correlation between the duration of MVPA and lower extremity muscle thickness as they did not engage in vigorous-intensity competitive sports and were more susceptible to the influence of the intensity and frequency of physical activity. MVPA refers to activities of daily living and exercise at an intensity of ≥3 MET, such as walking at a moderate speed or jogging (31). A previous study demonstrated that the vertical and anterior-posterior center-of-mass accelerations of the vastus lateralis muscle during gait increase with increasing gait speed that was the muscle activity of the vastus lateralis muscle increases with increasing gait speed (32). Furthermore, previous studies have reported a significant positive association between maximum walking speed and lower limb muscle strength (23,33), and lower limb muscle mass and lower limb muscle strength are closely related to walking function (34). A previous study demonstrated the association between running speed and the electromyography of lower limb muscle mass, reporting that the activity of the vastus lateralis exhibited the greatest increase in the pre-contact and braking phases with the increasing running speed (35). Previous studies have shown that walking and running movements associated with MVPA affect vastus lateralis muscle thickness in the lower extremities (23,31,33,35). The present study demonstrated that vastus lateralis muscle thickness was associated with the time spent performing moderate0 to vigorous-intensity activity in the group without regular exercise habits. Thus, these results suggest that the walking and running movements associated with the time spent performing MVPA may have increased the muscle activity of the vastus lateralis muscle and thickened in healthy young collegiate women without regular exercise habits.

The present study has some limitations which should be mentioned. First, the number of participants in the study was small and the study was limited to collegiate healthy young women attending the same university. Therefore, there was potential selection bias in the present study, and it is unclear whether these results are applicable to young males and other populations. Second, it was not possible to determine the causal association between physical activity levels and lower limb muscle thickness in collegiate healthy young women as the present study design was a cross-sectional study. Finally, the physical activity levels during sports activities were not evaluated using the accelerometer in the group with regular exercise habits, possibly leading to an underestimation of the physical activity levels. In addition, the limitations of the accelerometer include the impossibility of measuring activity while swimming or bathing, difficulty in evaluating upper limb movement, and the inability to determine physical activity levels, while moving such as bicycling. Therefore, the inability to measure these activities may have introduced errors when evaluating energy expenditure and time spent in physical activity.

Another limitation to the present study is the lack of dietary analyses that may be related to lower limb muscle thickness and IntraMAT. A previous study compared the association between IntraMAT and diet in older and younger women and found an association between macronutrients and alcohol intake (36). Therefore, it is possible that differences in composition due to diet may influence lower limb muscle thickness and IntraMAT.

However, few studies have evaluated muscle thickness in young women using ultrasound tomography (15). The results of the present study suggest that time spent preforming MVPA may be associated to lower-limb muscle thickness in female college students without regular exercise habits. The results presented herein suggest that continuous physical activity at moderate to vigorous intensity may contribute to maintaining and improving lower limb muscle thickness in young women. However, further studies with a greater number of participants, addressing the current limitations, and conducting longitudinal analyses are required to clarify the association between physical activity levels and lower limb muscle thickness and IntraMAT in healthy young collegiate women, as well as the influence of diet.

In conclusion, the present study examined the association of physical activity with lower limb muscle thickness, IntraMAT and muscle strength. No significant association between the physical activity level and muscle thickness or strength was observed in the group with regular exercise habits. However, in participants without regular exercise habits, physical activity levels, particularly the time spent performing moderate- to vigorous-intensity activity, were positively associated with vastus lateralis muscle thickness per thigh length and also with a higher echo intensity of the rectus femoris (a.u.). These results suggest that the presence or absence of an exercise habit and a reduction in the time spent in MVPA may affect lower limb muscle thickness and IntraMAT in healthy young collegiate women without regular exercise habits. Therefore, the lifestyle guidance of a reduction in the time spent in physical inactivity and continued time spent in MVPA is necessary to maintain skeletal muscle mass and to decrease in IntraMAT in young women.

## Figures and Tables

**Table I:** Physical characteristics of all of the study participants.

Characteristic	Value
Age (years)	20.7±1.0
Height (cm)	158.9±5.1
Weight (kg)	57.4±7.6
BMI (kg/m^2^)	22.8±3.2
Waist circumference (cm)	75.8±7.6
Hip circumference (cm)	93.7±6.3
Physical activity	
Total energy expenditure (cal/day)	1791.4±152.7
Number of step counts (steps/day)	7599.6±2338.9
Time spent in light intensity (min/day)	50.3±17.2
Time spent in moderate to vigorous intensity (min/day)	25.2±11.8
Muscle thickness	
Rectus femoris (cm)	1.72±0.32
Vastus intermedius of the anterior sites (cm)	1.47±0.43
Rectus femoris + vastus intermedius of the anterior site (cm)	3.20±0.70
Echo intensity of rectus femoris (a.u.)	64.62±9.83
Vastus lateralis (cm)	2.11±0.30
Vastus intermedius of the lateral sites (cm)	1.80±0.34
Vastus lateralis + vastus intermedius of the lateral site (cm)	3.91±0.49
Echo intensity of vastus lateralis (a.u.)	56.51±8.74
Rectus femoris/thigh length	0.05±0.01
Vastus intermedius of the anterior sites/thigh length	0.04±0.02
Rectus femoris + vastus intermedius of the anterior site/thigh length	0.10±0.03
Vastus lateralis/thigh length	0.06±0.01
Vastus intermedius of the lateral sites/thigh length	0.05±0.01
Vastus lateralis + vastus intermedius of the lateral site/thigh length	0.12±0.02
Muscle strength	
MVC force during isometric knee extension/weight (Nm/kg)	2.79±0.50

Data are expressed as the mean ± SD. BMI, body mass index; MVC, maximal voluntary contraction.

**Table II:** Correlation of total energy expenditure and time spent in physical activity with muscle thickness and muscle strength in all the study participants.

	Total energy expenditure		Number of step counts		Time spent in light intensity		Time spent in moderate to vigorous intensity	
Parameter	R	P-value	R	P-value	R	P-value	R	P-value
Rectus femoris/thigh length	0.307	0.188	-0.021	0.930	-0.130	0.586	0.159	0.502
Vastus intermedius of the anterior sites/thigh length	0.341	0.141	-0.011	0.964	-0.060	0.802	0.084	0.725
Rectus femoris + vastus intermedius of the anterior site/thigh length	0.343	0.139	-0.016	0.947	-0.094	0.694	0.121	0.610
Echo intensity of rectus femoris (a.u.)	-0.002	0.994	0.064	0.787	0.212	0.370	-0.113	0.635
Vastus lateralis/thigh length	0.251	0.287	0.339	0.144	0.274	0.242	0.204	0.387
Vastus intermedius of the lateral sites/thigh length	0.130	0.585	0.043	0.856	0.009	0.969	0.145	0.543
Vastus lateralis + vastus intermedius of the lateral site/thigh length	0.217	0.359	0.203	0.390	0.147	0.536	0.203	0.391
Echo intensity of vastus lateralis (a.u.)	0.007	0.977	-0.122	0.608	-0.272	0.246	0.118	0.620
MVC force during isometric knee extension/weight (Nm/kg)	-0.124	0.604	-0.075	0.754	0.014	0.955	-0.095	0.691

Data are expressed as correlation coefficients and P-values. MVC, maximal voluntary contraction.

**Table III:** Differences in physical characteristics between the groups with and without regular exercise habits.

Morphometrics	Without habit group (n=12)	Exercise habit group (n=8)	P-value
Age (years)	20.9±1.2	20.3±0.5	0.111
Height (cm)	158.8±5.9	159.2±4.2	0.858
Weight (kg)	55.6±7.0	60.2±8.1	0.213
BMI (kg/m^2^)	22.0±2.3	23.9±4.2	0.287
Waist circumference (cm)	75.5±7.4	76.4±8.3	0.797
Hip circumference (cm)	92.6±5.9	95.2±6.9	0.398
Physical activity			
Total energy expenditure (kcal/day)	1759.1±162.5	1839.9±131.7	0.239
Number of step counts (steps/day)	7542.1±2549.9	7685.9±2148.5	0.893
Time spent in light intensity (min/day)	50.0±18.2	50.9±16.8	0.914
Time spent in moderate to vigorous intensity (min/day)	24.6±10.5	26.2±14.3	0.787
Muscle thickness			
Rectus femoris/thigh length	0.05±0.01	0.06±0.01	0.009
Vastus intermedius of the anterior sites/thigh length	0.04±0.01	0.06±0.02	0.025
Rectus femoris + vastus intermedius of the anterior site/thigh length	0.08±0.02	0.12±0.03	0.011
Echo intensity of rectus femoris (a.u.)	67.84±10.16	59.78±7.43	0.056
Vastus lateralis/thigh length	0.06±0.01	0.06±0.01	0.620
Vastus intermedius of the lateral sites/thigh length	0.05±0.01	0.06±0.01	0.126
Vastus lateralis + vastus intermedius of the lateral site/thigh length	0.11±0.02	0.12±0.02	0.437
Echo intensity of vastus lateralis (a.u.)	56.65±9.04	56.31±8.88	0.936
Muscle strength			
MVC force during isometric knee extension/weight (Nm/kg)	2.52±0.41	3.19±0.32	<0.001

Data are expressed as the means ±SD. BMI, body mass index; MVC, maximal voluntary contraction.

**Table IV:** Correlation of total energy expenditure and time spent in physical activity with muscle thickness, and muscle strength in groups with and without regular exercise habits.

	Total energy expenditure				Number of step counts				Time spent in light intensity				Time spent in moderate to vigorous intensity			
	Group without exercise		Group with exercise habits		Group without exercise		Group with exercise habits		Group without exercise		Group with exercise habits		Group without exercise		Group with exercise habits	
Parameter	R	P-value	R	P-value	R	P-value	R	P-value	R	P-value	R	P-value	R	P-value	R	P-value
Rectus femoris/thigh length	0.288	0.475	0.136	0.748	-0.157	0.626	0.147	0.729	-0.140	0.663	-0.238	0.570	-0.006	0.985	0.318	0.443
Vastus intermedius of the anterior sites/thigh length	0.077	0.812	0.439	0.276	-0.128	0.693	0.041	0.923	-0.111	0.732	-0.091	0.831	-0.016	0.961	0.094	0.825
Rectus femoris + vastus intermedius of the anterior site/thigh length	0.161	0.617	0.361	0.380	-0.148	0.646	0.085	0.841	-0.131	0.685	-0.155	0.714	-0.011	0.972	0.188	0.656
Echo intensity of rectus femoris (a.u.)	0.249	0.435	-0.212	0.614	0.207	0.519	-0.225	0.592	0.276	0.384	0.174	0.680	0.055	0.864	-0.338	0.413
Vastus lateralis/thigh length	0.211	0.511	0.463	0.248	0.580	0.048	-0.096	0.822	0.305	0.335	0.235	0.575	0.665	0.018	-0.302	0.467
Vastus intermedius of the lateral sites/thigh length	0.029	0.929	0.051	0.905	0.091	0.777	-0.059	0.890	0.058	0.859	-0.087	0.837	0.293	0.355	-0.039	0.927
Vastus lateralis + vastus intermedius of the lateral site/thigh length	0.130	0.687	0.265	0.526	0.366	0.242	-0.087	0.838	0.198	0.536	0.060	0.889	0.537	0.072	-0.177	0.675
Echo intensity of vastus lateralis (a.u.)	0.049	0.880	-0.060	0.888	-0.306	0.333	0.228	0.587	-0.243	0.447	-0.322	0.436	-0.193	0.548	0.489	0.219
MVC force during isometric knee extension/weight (Nm/kg)	-0.466	0.127	-0.315	0.447	-0.112	0.729	-0.712	0.683	-0.070	0.830	0.132	0.755	-0.156	0.628	-0.263	0.529

Data are expressed as correlation coefficients and P-values. MVC, maximal voluntary contraction.

## Data Availability

The data generated in the present study may be requested from the corresponding author.
